# Association of Blood Subgroups With PCR Test Positivity and Lung Involvement in Patients With COVID-19

**DOI:** 10.7759/cureus.14172

**Published:** 2021-03-29

**Authors:** Yonca Coluk, Omer Hizli, Selda Gunaydın, Guven Yildirim, Elif Baysal, Guliz Ozgen Hergul

**Affiliations:** 1 Department of Otolaryngology, Giresun University, Faculty of Medicine, Giresun, TUR; 2 Department of Otolaryngology, Balikesir University, Faculty of Medicine, Balikesir, TUR; 3 Department of Pulmonary Medicine, Giresun University, Giresun, TUR; 4 Department of Psychiatry, Giresun University, Faculty of Medicine, Giresun, TUR

**Keywords:** blood groups, covid-19, sars-cov-2, lung involvement

## Abstract

Objective

The goal of this study was to investigate whether blood group type caused susceptibility to COVID-19 infection.

Methods

Two hundred and eleven consecutive patients admitted with various symptoms associated with COVID-19 were included. We compared the AB0 and Rh subgroup distributions between patients with a positive polymerase chain reaction (PCR) test result and the patients without. We compared the AB0 and Rh subgroup distributions between patients with lung involvement and patients without. Additionally, comparisons were performed between the patients both with positive PCR result and lung involvement, and the patients with a negative PCR result.

Results

No significant difference of ABO and Rh subgroup distributions was evident between patients with and without a positive PCR test result (p=0.632 and p=0.962). No significant difference of ABO and Rh subgroup distributions was evident between the patients with and without lung involvement (p=0.097 and p=0.797). No significant difference of ABO and Rh subgroup distributions was evident among patients both with PCR positivity and lung involvement, patients with only PCR positivity, and the patients with negative PCR test results (p=0.3 and p=0.993).

Conclusion

All blood group types seem to have an equal risk of COVID-19 infection. Everyone should follow the precautions to avoid the COVID-19 infection.

## Introduction

Coronaviruses are a large family of viruses causing mild, self-limiting infections common in the general population, like common cold, also more serious infections such as middle east respiratory syndrome (MERS) and severe acute respiratory syndrome (SARS) [1]. The coronavirus disease-2019 (COVID-19) caused by SARS-CoV-2 subtype of coronavirus was declared as a pandemic in March 2020 by World Health Organization [2].

Risk factors that may create susceptibility to COVID-19 were investigated in various studies. Comorbidities such as respiratory and cardiovascular disease, advanced age and male gender are known to be the main risk factors increasing the susceptibility to Covid-19 and the severity of the disease [3]. The effects of ABO and Rh blood subgroup differences on the characteristic of the disease in patients with COVID-19 are still a matter of debate. In the prior literature, there exist a limited number of studies investigating the association of COVID-19 with blood groups [4-7].

The goal of this retrospective, cross-sectional study was to investigate the blood group distribution of COVID-19 patients and to determine whether any blood group type caused a susceptibility to this viral infection.

## Materials and methods

Patients, groups, and study design

This study was conducted in accordance with the dictates of World Health Organization- Declaration of Helsinki, after providing the official permission of Turkish Ministry of Health, Scientific Research Committee for COVID-19, with the informed consent of the patients and approval of the local ethical committee (IRB Number: 22.05.2020/17). Two hundred and eleven consecutive patients admitted to the outpatient clinic of COVID-19 with various symptoms that might be associated with COVID-19 were included in the study. Medical information of all patients including age, gender, PCR test results, lung involvement status (detected by thorax CT), ABO and Rh blood subgroups were recorded retrospectively. Excluded from the study were only the patients under the age of 18 years.

**Figure 1:**
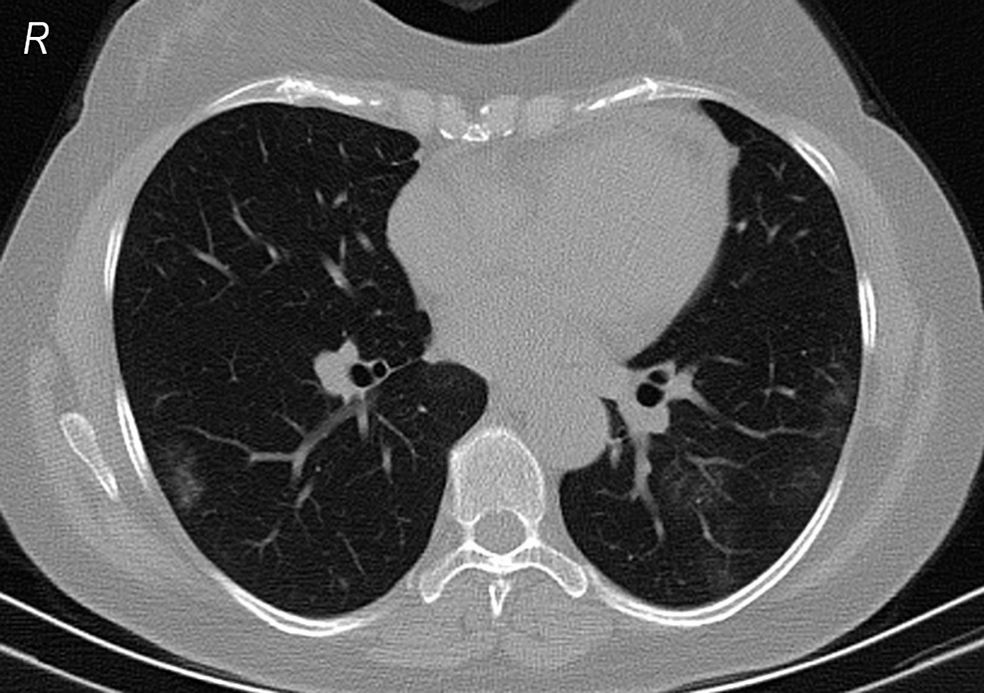
Lung involvement in a 46-year-old woman with a positive PCR test result. PCR: polymerase chain reaction.

**Figure 2:**
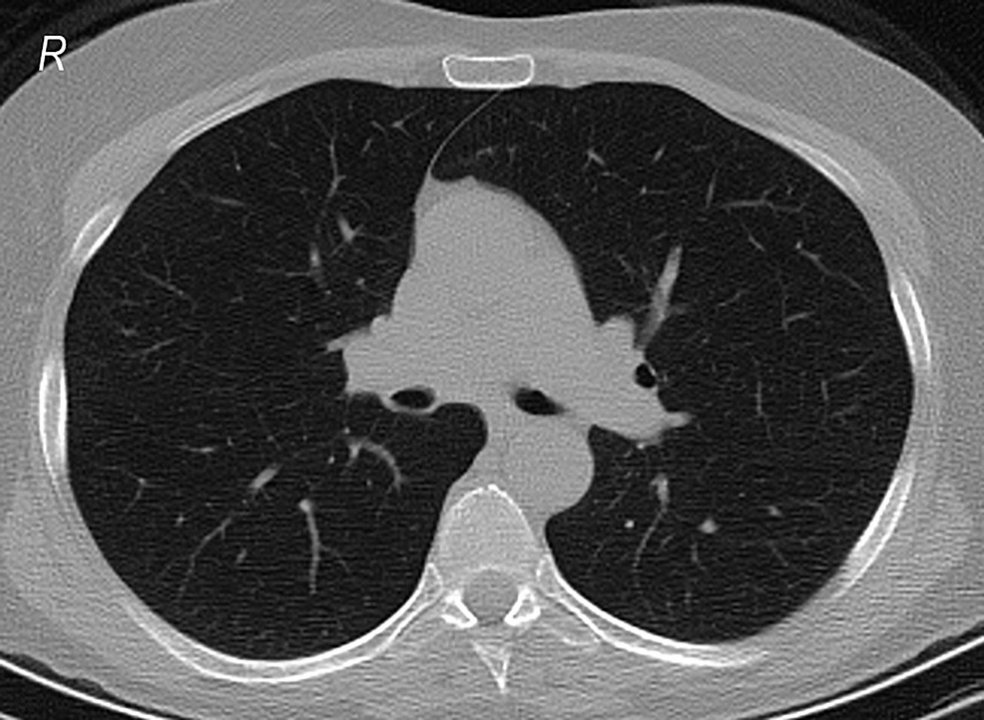
A CT radiograph of a 61-year-old woman without CT findings and with a positive PCR test result. PCR: polymerase chain reaction.

Thorax CT investigations were performed using a 16-slice CT unit (Somatom Emotion; Siemens, Munich, Germany) with Picture Archiving Communication Systems (PACS) (Akgun, Ankara, Turkey). The patients with any specific CT finding of COVID-19 were considered as having a lung involvement. The PCR tests for detecting COVID-19 were performed using Biospeedy® COVID-19 qPCR Detection Kit (Bioeksen R&D Technologies Ltd., Istanbul, Turkey). We constituted a variety of study groups for the comparison of ABO blood subgroups and Rh subgroups of the patients with COVID-19, to determine whether an association of blood subgroups was present with PCR positivity and lung involvement. First, we compared the ABO and Rh subgroup distributions between the patients with a positive PCR test result and the patients without (Figures 1, 2).

**Figure 3:**
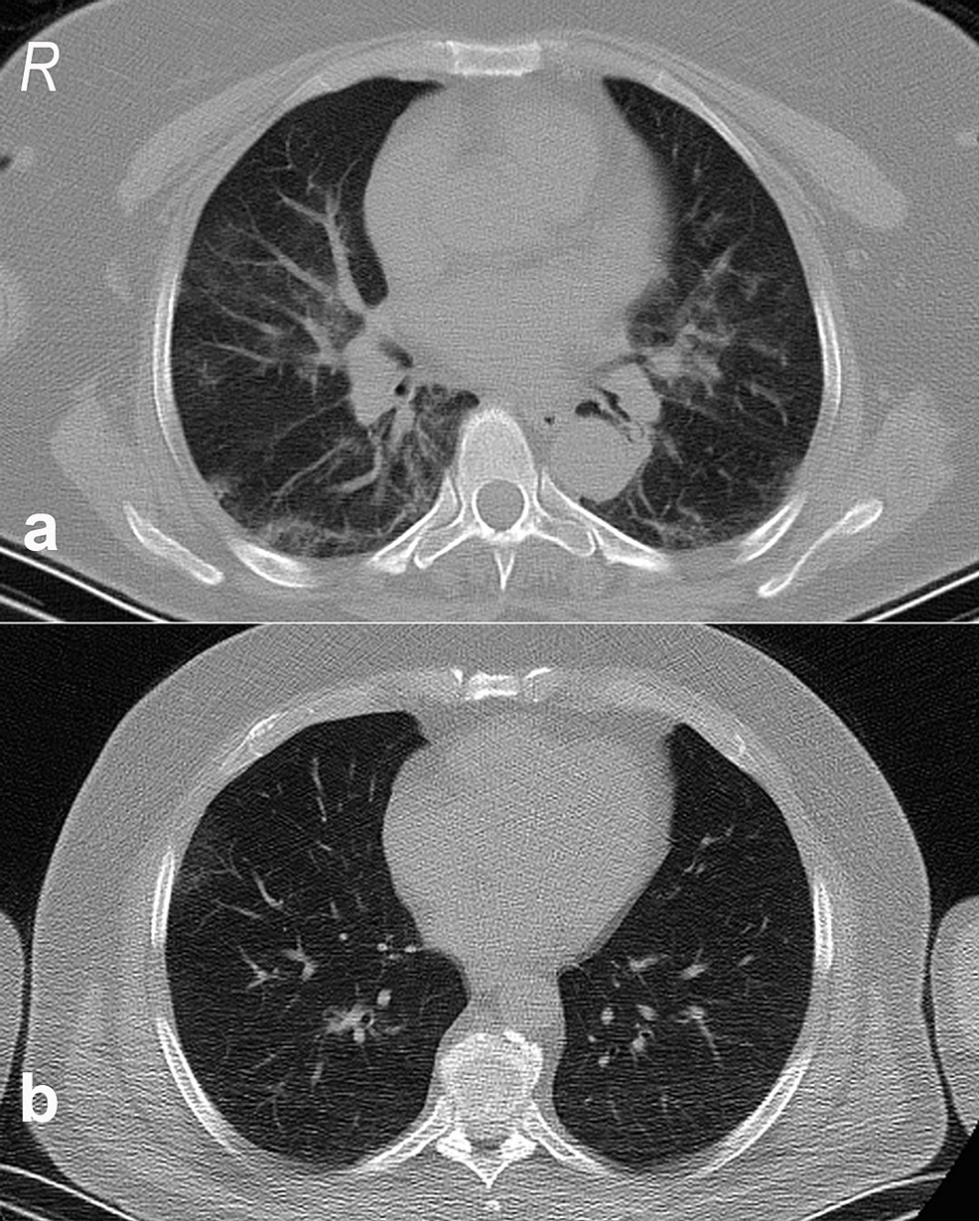
(a) Specific thorax CT findings of a 63-year-old woman with a negative PCR test result. (b) Specific thorax CT findings of a 57-year-old man with a positive PCR test result. PCR: polymerase chain reaction.

Then, we compared the ABO and Rh subgroup distributions between the patients with lung involvement and the patients without (Figure 3). Last, the comparisons were performed between the patients both with positive PCR result and lung involvement, and the patients with a negative PCR result.

Statistical analysis

Results were presented as number (percentage). For the comparisons of the blood subgroup distributions, Chi- square test was used. The cross-tab analysis results were presented in tables. Statistical analysis was performed using SPSS 23.0 software for Windows (SPSS Inc., Chicago, IL). A p-value under 0.05 was considered statistically significant.

## Results

Among 211 patients totally included, 144 (68.25%) had a positive PCR test result and 67 (38.75%) had a negative PCR test result. While 154 (73%) patients had a lung involvement, 57 (27%) patients had no finding in thorax CT sections. The number of the patients both with positive PCR result and lung involvement was 97 (46%).

Table 1 presents the ABO subgroup distributions, and Table 2 presents the Rh subgroup distributions of the patients with and without PCR positivity. Chi- square analysis revealed that no significant difference of ABO and Rh subgroup distributions was evident between the patients with and without a positive PCR test result (p=0.632 and p=0.962, respectively).

**Table 1:** Comparison of ABO subgroup distributions of the patients with and without PCR positivity. p=0.632. PCR: polymerase chain reaction.

		ABO blood subgroup				Total
		A	B	O	AB	
PCR	Negative	32 (47.7%)	12 (17.9%)	19 (28.4%)	4 (6%)	67 (100%)
	Positive	69 (47.9%)	20 (13.9%)	50 (34.7%)	5 (3.5%)	144 (100%)
Total		101 (47.9%)	32 (15.2%)	69 (32.7%)	9 (4.2%)	211 (100%)

**Table 2:** Comparison of Rh subgroup distributions of the patients with and without PCR positivity. p=0.962. PCR: polymerase chain reaction.

		Rh blood subgroup		Total
		Negative	Positive	
PCR	Negative	9 (13.4%)	58 (86.6%)	67 (100%)
	Positive	19 (13.2%)	125 (86.8%)	144 (100%)
Total		28 (13.3%)	183 (86.7%)	211 (100%)

Table 3 presents the ABO subgroup distributions, and Table 4 presents the Rh subgroup distributions of the patients with and without lung involvement. Chi-square analysis revealed that no significant difference of ABO and Rh subgroup distributions was evident between the patients with and without lung involvement (p=0.097 and p=0.797, respectively).

**Table 3:** Comparison of ABO subgroup distributions of the patients with and without lung involvement. p=0.097.

	ABO blood subgroup				Total
	A	B	O	AB	
Patients without lung involvement	21 (36.8%)	12 (21%)	23 (40.4%)	1 (1.8%)	57 (100%)
Patients with lung involvement	80 (51.9%)	20 (13%)	46 (29.9%)	8 (5.2%)	154 (100%)
Total	101 (47.9%)	32 (15.2%)	69 (32.7%)	9 (4.2%)	211 (100%)

**Table 4:** Comparison of Rh subgroup distributions of the patients with and without lung involvement. p=0.797.

	Rh blood subgroup		Total
	Negative	Positive	
Patients without lung involvement	7 (12.3%)	50 (87.7%)	57 (100%)
Patients with lung involvement	21 (13.6%)	133 (86.4%)	154 (100%)
Total	28 (13.3)	183 (86.7%)	211 (100%)

Table 5 presents the ABO subgroup distributions, and Table 6 presents the Rh subgroup distributions of patients both with PCR positivity and lung involvement, in comparison with the patients with only PCR positivity (without long involvement), and the patients with negative PCR test results. Chi-square analysis revealed that no significant difference of ABO and Rh subgroup distributions was evident among these three groups (p=0.3 and p=0.993, respectively).

**Table 5:** Comparison of ABO subgroup distributions of three groups. p=0.3. PCR: polymerase chain reaction.

ABO blood subgroup	PCR and lung involvement status			Total
	PCR (-)	PCR+CT finding (-)	PCR+CT finding (+)	
A	32 (47.8%)	17 (36.2%)	52 (53.6%)	101 (47.9%)
B	12 (17.9%)	10 (21.3%)	10 (10.3%)	32 (15.1%)
O	19 (28.4%)	19 (40.4%)	31 (32%)	69 (32.7%)
AB	4 (5.9%)	1 (2.1%)	4 (4.1%)	9 (4.3%%)
Total	67 (100%)	47 (100%)	97 (100%)	211 (100%)

**Table 6:** Comparison of Rh subgroup distributions of three groups. p=0.993. PCR: polymerase chain reaction.

Rh blood subgroup	PCR and lung involvement status			Total
	PCR (-)	PCR+CT finding (-)	PCR+CT finding (+)	
Negative	9 (13.4%)	6 (12.8%)	13 (13.4%)	28 (13.3%)
Positive	58 (86.6%)	41 (87.2%)	84 (86.6%)	183 (86.7%)
Total	67 (100%)	47 (100%)	97 (100%)	211 (100%)

## Discussion

This study was conducted with a total of 211 patients with COVID-19, 144 of whom were confirmed with positive PCR test results obtained by specimens from nasal cavity and nasopharynx and the rest showed specific lung involvement on thorax computed tomography. According to our results there was no significant difference in ABO and Rh subgroup distributions between the patients with and without a positive PCR test result, and between the patients with and without lung involvement. Additionally, patients both with PCR positivity and lung involvement did not show a significantly different blood type distribution compared both to the patients with only PCR positivity (without lung involvement) and the patients with negative PCR test results.

AB0 blood group system antigens including A, B and H determinants are composed of complex carbohydrate molecules expressed on red blood cells and on many other cell surfaces/tissues such as epithelial cells, sensory neurons, platelets and endothelia of blood vessels [8]. Guillon et al. showed that human natural anti-A antibodies found in individuals with blood group O blocked the interaction between virus and its receptor, by inhibiting the adhesion of S spike protein of SARS-CoV-2 and interrupting the angiotensin-converting enzyme-2 expressing cell line [9]. According to this report, blood group AB that do not have anti-A antibody might have a higher susceptibility to this infection like blood group A, but there does not exist any direct evidence in the literature to show that predisposition [10].

Many studies aiming to reveal the association between the ABO blood groups and various diseases revealed that ABO system might play an important role in the pathogenesis of immunological, cardiovascular, and neoplastic diseases [11,12]. ABO blood group system antibodies are a part of innate immune system and play role in the fight against some parasites, bacteria, and enveloped viruses [13,14]. In previous publications, certain blood group antigen expression was reported to alter host susceptibility to some infectious diseases. Association between ABO blood group antigens and specific pathogens such as Norwalk virus, dengue virus, hepatitis B virus and rotavirus was shown [4-7]. Furthermore, it was suggested that different prevalence of ABO blood group genotypes among various populations could be associated with selective pressure of some infectious diseases, particularly of the infections with Plasmodium falciparum and Vibrio cholera [8,15].

Since the beginning of the SARS-CoV-2 pandemic, several studies have been conducted to investigate the relationship between blood groups and the risk and the frequency of infection. The first of these studies showed that blood group A was significantly higher in COVID-19 patients compared to the control group and blood group O in COVID-19 patients was significantly lower compared to the control group [16]. Similarly, Cheng et al. reported that those with blood group O had a lower frequency of SARS-CoV infection than the non-O groups during the SARS epidemic [17]. Afterwards, in studies conducted in various centers, it was shown that blood group A was associated with an increased risk of SARS-CoV-2 infection and blood group 0 had lower susceptibility to SARS-CoV-2 infection [18-20]. Zhao et al. also mentioned that blood group A was linked to higher mortality risk in discordance with blood group O [16].

Dzik et al. reported that percentage of individuals with blood group 0 was non-significantly and slightly higher in the population of Massachusetts and Boston. They explained this result by the Hispanic race dominancy of Boston, since blood group 0 was seen more frequently in Hispanics [21,22].

Goker et al. presented that blood group A was significantly more frequent and the blood group 0 was less frequent amongst 186 Turkish COVID-19 patients [20]. However, the present study demonstrates that ABO and Rh subgroup distributions are similar among the Turkish COVID-19 patients with and without a positive PCR test result and lung involvement. According to our results, there was no association of susceptibility to COVID-19 infection with ABO blood groups, among Turkish people. It may be considered that actual results in the literature might vary in different races and countries depending on the differences in blood group distribution. Thus, the blood group distribution of the patients with COVID-19 might be reflecting the distribution of the normal population. If the normal population has a specific blood group dominancy, patients with COVID-19 may have the same blood group dominancy as well.

The major limitation of this retrospective study was the small number of patients because the majority of patients diagnosed with COVID-19 did not have a recorded blood subgroup in their medical records, thus we included only the patients with a recorded blood subgroup.

## Conclusions

In conclusion, people with all blood group types have an equal risk of COVID-19 infection. Everyone should follow the precautions to avoid the COVID-19 infection. However, underlying molecular mechanism of the relationship between the blood groups and the infection needs further molecular studies and larger multi-center research with individuals of different ethnicities. Revealing this linkage of ABO and Rh system to the prevalence and mortality of COVID-19 is important in terms of understanding both the pathophysiology of the disease and the convalescent plasma therapy.
